# Miscellaneous Notes

**Published:** 1880-02

**Authors:** Edgar D. Swain


### MISCELLANEOUS NOTES.

---

In the province of Sze-chuen, China, the production of white wax from an insect attaching itself to the twigs of the *Ligustrum Lacidum*, an ever-green tree, reaches an annual value of over $3,000,000.——A writer in the *American Ship* says that "nearly all the steamers navigating the Mississippi and its tributaries, are constructed upon the Ohio."——A London writer, comparing the growing popularity of American manufactures with the corresponding decline of the English, says it is due to the reliable average quality of the former and the deterioration of the latter.——The principal supply of water for irrigation in Utah Territory, comes from "the snows which fall in the mountains and remain there during the summer."——Modern science has made carbon one of the corner-stones of civilization.— With the beginning of the new year, the *Cincinnati Commercial* commenced the publication of a daily weather map.——Charles Francis Adams, Jr., is the author of the first work treating of railway casualties; he calls it "Notes on Railway Accidents."—— The *Popular Science Monthly* for January contains a most excellent article on the International Weather Service, from the pen of Prof. L. B. Maury.——As a result of investigation, it is found that one man in every 125 employed upon the German State Railways, is affected by color-blindness.——Profs. Klebs and Tomasi have discovered a microscopic fungus which appears to be the physical cause of intermittent fever. It was found in the atmosphere and soil in the Agro Romano, the spores are ovate in form, movable and possess some lustre.——Samuel Sexton, M. D., of New York City, received the prize from the New York County Medical Society, for his essay " On affections of the Ear arising from Diseases of the Teeth." Dr. Sexton is an Ohio man.——Dr. Javal, a French opthalmologist says that myopia is caused by a " prolonged application of sight during childhood, combined with insufficient light."——Prof. Estlander thinks the entire parotid gland may be removed without serious detriment to the patient, as some power over the sphincter of that side is still retained.——The London *Lancet* is authority for the statement that " the truth would be that what we call cold-taking, is the result of a sufficient impression of cold to reduce the vital energy of nerve centers, presiding over the functions in special organs." The effects of a chill are to restore the vital energy of the nerve centers; and there is no more potent influence by which to attain this object, than a strong and sustained effort of the will. The man who resolves not to take cold, seldom does.——The work of the heart in twenty-four hours, is equal to nearly 125 feet tons.——A private letter to the editor, dated Vienna, Dec. 25th, 1879, states that Hebra, the great dermatologist, is ill and unable to lecture any more; his place being supplied by his son-in-law, Prof. Kaposi.——A new monthly, called the *Practitioner*, devoted to medical, surgical, obstetrical and dental science, the first number of which appeared in January, is published in Baltimore and edited by H. L. Byrd, A. M., M. D. and B. M. Wilkerson, D. D. S., M. D.——Letters from Herbert Spencer, announce his intention to cut short his journey in Egypt. His health has improved, and he intends to return in February.—*Cincinnati Commercial*, Jan. 18.——Robert Sattler, M. D., lecturer on opthamology in the Miami Medical College Cincinnati, is delivering a series of lectures upon the progressive development of the nervous system. His remarks are illustrated by photographic views projected upon a large screen, from a powerful magic lantern.—-Hon. T. W. Palmer, speaking of Yankees, says: "Firm in intent, but flexible as to methods and fertile in resource, the typical Yankee of to-day is the man who, more than another, puts himself in accord with natural laws.—- In 1879, 21,421 vessels arrived at New York, an increase of 2311 over 1878.——Remains of a pre-historic race of men are being brought to light in Kansas, and the evidence seems to show that, compared with their antiquity, the mound-builders are modern. —-All the gold in the world would make a pile only 25 feet wide, 45 feet long, and 25 feet high.—*Scientific American.*——The new German cure for phthisis consists of two inhalations daily of a 5 per cent. solution of benzoate of soda from Sigle's atomizing inhaler. The salt is supposed to destroy the specific bacteria to which the tuberculizing process is due.——English surgeons are now using the electric light during surgical operations when the weather is foggy.——Small-pox has reappeared in the United States, dating from the middle of November last. *Thorough vaccination is the only remedy* to prevent the spread of this loathsome disease.——Burlington, Iowa, is the healthiest city in the Union, the annual death rate being only 4.84 deaths per 1000 inhabitants. No wonder the *press* there, looks after city officials with a *Hawkeye.*

